# Neurorights vs. neuroprediction and lie detection: The imperative limits to criminal law

**DOI:** 10.3389/fpsyg.2022.1030439

**Published:** 2022-12-08

**Authors:** José Manuel Díaz Soto, Diego Borbón

**Affiliations:** ^1^Department of Criminal Law and Criminology, Universidad Externado de Colombia, Bogotá, Colombia; ^2^NeuroRights Research Group, The Latin American Observatory of Human Rights and Enterprises, Universidad Externado de Colombia, Bogotá, Colombia

**Keywords:** neurorights, neuroethics, neurolaw, neuroscience, neurotechnologies, human rights, criminology, neuromarkers

This paper analyzes the suitability of neurorights to limit the use of neuroprediction and lie detection neurotechnologies. We argue that some of their applications in criminal proceedings should be prohibited as they are severely intrusive to mental privacy and contrary to the dignity of the person. In that sense, we discuss whether neurorights can offer greater protection than current fundamental rights. We suggest that, as they have been conceived, neurorights may offer reduced protection and they should be framed to offer a true limit to the substantial barrier that is our mind and dignity. On the other hand, current human rights should be interpreted in such a way as to respect the dignity of the accused in criminal proceedings.

## A brief overview on neurorights

Since 2017, an innovative discussion framework has been created to protect people from potential abusive uses of neurotechnologies. Based on neuroethics, researchers Ienca and Andorno (2017) propose to create four neuro-specific human rights: cognitive liberty, psychological continuity, mental privacy, and integrity. Likewise, Yuste et al. (2017), and nowadays the NeuroRights Foundation, promotes the creation of five NeuroRights: the right to free will, mental privacy, personal identity, fair access to mental augmentation, and protection from bias (NeuroRights Foundation., 2022).

Furthermore, the reception of those initiatives has been such that on April 12, 2021, the Chilean Congress approved a Constitutional Reform endorsing the rights to physical and mental integrity (IACHR., 2022a).^1^

More recently, in the United Nations, resolution A/HRC/51/L.3 on neurotechnology and human rights was adopted (Human Rights Council HCR-UN, 2022).^2^

## Neuroprediction and lie detection

Research in neurotechnology is allowing us to have a better understanding of the brain and enabling technology for new treatment options and better quality of life (Stieglitz, 2021). On the other hand, in clinical translational science, neuroscientists are looking to apply this technology to assess, treat, and better understand complex socioemotional processes that underlie many forms of psychopathology (White et al., 2015).

In that direction, criminal justice systems are not far behind as criminal law cares about human behavior and specially the mind (Greely and Farahany, 2018). Some authors argue that neuroscience has the power to change the criminal justice systems, and that society would benefit from active collaboration between sciences (Altimus, 2017). Although neurotechnologies could provide useful tools for the judicial system, some pose numerous ethical challenges that hinder their implementation (Coronado, 2021; Borbón and Borbón, 2022).

Neuroprediction and lie detection neurotechnologies are a clear example of why it has become so essential to discuss neurorights. Neuroprediction comprises the use of structural or functional variables of the brain for medical and behavioral predictions (Morse, 2015). In recent years, artificial intelligence (A.I.) and neurotechnologies are being used to improve the accuracy of risk assessment tools (Kehl et al., 2017; Kiehl et al., 2018; Tortora et al., 2020), using neuroimaging data to predict recidivism and criminal behavior (Aharoni et al., 2013; Kiehl et al., 2018; Delfin et al., 2019). In that sense, findings in neurocriminology have managed to identify structural and functional deficits in the brain and their relationship with antisocial behavior (Bellesi et al., 2019; Katzin et al., 2020; Ruiz and Muñoz, 2021; Borbón, 2022). These empirical results could be used by neuroprediction algorithms to identify the neuro markers that influence deviant behavior.

Parallel to advances in neuroprediction, the use of neurotechnologies for lie detection has been explored. Recent efforts to detect lies have focused on measures in the brain, believing that these may be more reliable than physiological responses in other parts of the body. Some studies used tools such as positron emission tomography, electroencephalography, functional near-infrared spectroscopy, and functional magnetic resonance imaging (Norman et al., 2006; Greely and Illes, 2007; Abootalebi et al., 2009; Langleben and Moriarty, 2013; Farah et al., 2014; Li et al., 2018).

However, these technologies are far from perfect and remain open to the subjectivity of those who interpret the results obtained while being hardly validated or reliable (Greely, 2009; Lowenberg, 2010; Schauer, 2010). Furthermore, the deep ignorance that we still have about the brain, given that neuroscience is a developing science, implies that we must proceed with caution, far from the current “neuro hype” (Bigenwald and Chambon, 2019; Morse, 2019). Neuroprediction and lie detection are not able to offer proof standards of certainty, but only of probability. In this sense, its use to serve as evidence for prosecution, or even to extend the length of criminal sentences, should be strictly regulated.

## Neurorights: Progressivity and non-regressivity

In terms of human rights, the principle of progressivity is recognized in the preamble of the Universal Declaration of Human Rights and expressly protected by the Inter-American Human Rights System (IACHR., 1993). The principle of progressivity entails an obligation of non-regression, which implies that the progress made in the field of human rights is irreversible, it can always be expanded but never reduced (Cunego, 2016). Under that scope, the fundamental reason to create neurorights should be to offer citizens a greater scope of protection. However, some interpretations that could be extended to the initial proposal of neurorights concern us because they may end up transgressing these principles.

The initial paper by Ienca and Andorno (2017) proposes to create a right to cognitive liberty, which implies being able to reject neurotechnological applications in their negative facet. However, throughout the text they recognize that neurorights, like any of the current fundamental rights, are relative and that in certain circumstances they could be reduced substantially. Regarding the neuroright to mental privacy, they maintain that the collection, use, and disclosure of private information is permissible when the public interest is at stake (Ienca and Andorno, 2017). Also, considering the painless nature of brain scans, they suggest that there could be good reasons for thinking that their nonconsensual use would be justified, with a court warrant, under special circumstances (Ienca and Andorno, 2017). This, we believe, would include the debate on neuroprediction and lie detection. In addition, when dealing with a subject as sensitive as “moral enhancement”, Ienca and Andorno (2017) suggest that it is possible to argue on utilitarian grounds that violations of the right to mental integrity could be allowed for persistent violent offenders, but they prefer not to take a definitive position on that issue.

Our argumentation does not ignore that human rights in general are relative and that in the judicial practice they are weighted against other rights. On the contrary, we intend to bring the discussion closer to the insuperable principle and rule of human dignity, as well as to advocate for absolute prohibitions when neurotechnology is used against the person.

In that sense, revealing the neural correlates of individual thoughts and feelings can be seen as an intrusion into privacy. The violations of freedom would be even more evident in the uses of neuroprediction for sentencing or punitive purposes, or lie detection for prosecution. In those cases, the person is taken simply as a means or an object of a criminal proceeding. Should potential offenders of the Law be forced to undergo neuroimaging tests against their will, under the pretext of public safety? (Coppola, 2018). Faced with the risks implied by technological advances, and the increasingly intrusive mechanisms in privacy and the free decision of people, we advocate for the rigorous regulation of those coercive uses.

We think that inordinate reliance on neurotechnologies and A.I. could bias judicial decisions, and even put an end to the purpose of having a judicial system and criminal proceedings at all. We certainly agree that excessive and unreasonable reliance on those technologies should be avoided (Tortora et al., 2020). Proceeding in this way raises serious ethical implications (Nadelhoffer and Sinnott-Armstrong, 2012; Tortora et al., 2020) and would undermine the rights of the accused, the prohibition of self-incrimination, the presumption of innocence, the right to refuse medical treatment, to due process, defense and contradiction, the culpability principle and the *mens rea*. But especially, we consider that insisting on coercively implementing these technologies violates human dignity, a guiding principle in any democratic society that respects the rule of law. In the end, neurorights would not be complying with the principles of progressivity and non-regression. Instead, they would be turning into ambiguous clauses for the punitive power of the State.

## Between neurorights and human rights

Even when criminal law has limits, such as the weights imposed by fundamental rights, it always retains intrinsic brutality, which makes its moral legitimacy problematic and uncertain (Ferrajoli, 1995). This brutality would be exacerbated if the State acquires new neurotechnological tools for punitive purposes (Borbón and Borbón, 2022). In this direction, there is no doubt that neuroscientific progress must be regulated. However, what is not so clear is whether neurorights are the best alternative.

Bublitz (2022) has criticized the inflation of rights and their resulting devaluation. This author affirms that there has not been a real academic debate, nor has it been explained why the current rights are insufficient. Borbón and Borbón (2021) have presented arguments in that same direction affirming that the current human rights already protect freedom, consent, equality, integrity, privacy, and others. In this sense, they propose that it would be much more necessary to propose legal and conventional regulations that are much clearer, precise, and extensive (Borbón and Borbón, 2021). Likewise, Ienca (2021) asserts that the relatively sporadic presence of neurorights in the academic literature poses a risk of semantic-normative ambiguity and conceptual confusion; López and Madrid (2021) argue that the legal consequences would be disastrous if neurorights are normatively manifested in a frivolous or imprecise way; and Fins (2022) states that the current Chilean neurorights reforms are vague and premature.

## Conclusion and proposal

We propose further academic and political deliberation to reach a consensus on the legal instruments necessary to effectively regulate the advancement of neuroscience. Neurotechnologies used coercively for neuroprediction or lie detection should be extensively regulated or even prohibited if they are used for punitive purposes, criminal prosecution, and the limitation of freedom.

In this sense, the direction proposed by Ruiz and Muñoz (2021) seems relevant to us. They re-define neuroprediction into “neuroprevention”, assuming a non-reductionist position to reach an early detection of risk factors that allows timely interventions through the application of training practices in cognitive skills aimed at reducing criminogenic factors. In general, the intent is to balance public safety with a scientifically based opportunity to reintegrate the person into society (Ruiz and Muñoz, 2021).

On the other hand, we consider that human dignity is a solid foundation to build the legal regulations of neuroscience. For the Law, human dignity is a wideranging constitutional value, gathering a whole array of protections, benefits, structures, empowerments, entitlements, institutions, forms of respect, and equalizations going well beyond a list of individual rights (Waldron, 2019). On our view, human dignity is not ponderable since it will never be conventionally admissible to treat the other as a simple object or means. Rights in general can be subject to judicial interference and even be susceptible to profound limitations, except in the case of human dignity. For those same reasons, for example, it will never be valid to torture a criminal, even when States restrict other rights.

In this sense, to protect the substantive legal grounds of freedom, integrity, privacy, or equality, any legislative proposal must be based on the ever-valid principle of human dignity. We advocate for a strong concept of human dignity, which would imply a strict regulation on the use of neurotechnologies in criminal proceedings. This could be similar to the conventional prohibition of cruel, inhuman or degrading punishment.

All things considered, we maintain that, if neurorights are considered necessary, they should be enshrined in such a way that they prevent States from using technologies without the consent and for punitive purposes. In the same way, current fundamental rights, along with new specific and clear international treaties, must be aimed at guaranteeing the principle of human dignity. Neuroscience, in this sense, can be used in ways that respect people's rights, even as valid defensive strategies in criminal proceedings, with the person's due informed consent. In the end, our call is to firmly give the battle to preserve the most intimate personal corner: our mind.

## Author contributions

Both authors listed have made a substantial, direct, and intellectual contribution to the work and approved it for publication.

## Footnotes

^1^On September 4, 2022, the people of Chile voted to deny the constitutional referendum that sought to replace the 1980 Constitution. The denial of the referendum did not affect the reform of neurorights, since it was incorporated into article 19 of the old Constitution, which remains effective as of October 2022. At the moment, the Chilean Government is preparing a new constitutional convention whose results will again be voted on by the Chilean people.

^2^This resolution is a great step forward, as the Human Rights Council will now be in charge of studying the opportunities and challenges of neurotechnology in relation to the promotion and protection of human rights. It will also allow to know the opinions and contributions of the countries and of the academy. To learn about the most recent academic research on neurorights, we recommend consulting the recent Research Topic published with Frontiers (García-López et al., 2021), which includes important works on this subject (Borbón and Borbón, 2021; Ienca, 2021; Inglese and Lavazza, 2021; Larrivee, 2021; Schleim, 2021; Wajnerman, 2021). See also (Collecchia, 2021; Goering et al., 2021; Fyfe et al., 2022; Herrera-Ferrá et al., 2022; Vidal, 2022).
